# Mitochondrial Function and Mitophagy in the Elderly: Effects of Exercise

**DOI:** 10.1155/2017/2012798

**Published:** 2017-08-16

**Authors:** Osvaldo C. Moreira, Brisamar Estébanez, Susana Martínez-Florez, José A. de Paz, María J. Cuevas, Javier González-Gallego

**Affiliations:** ^1^Institute of Biomedicine (IBIOMED), University of León, León, Spain; ^2^Institute of Biological Sciences and Health, Federal University of Viçosa-Campus Florestal, Florestal, MG, Brazil

## Abstract

Aging is a natural, multifactorial and multiorganic phenomenon wherein there are gradual physiological and pathological changes over time. Aging has been associated with a decrease of autophagy capacity and mitochondrial functions, such as biogenesis, dynamics, and mitophagy. These processes are essential for the maintenance of mitochondrial structural integrity and, therefore, for cell life, since mitochondrial dysfunction leads to an impairment of energy metabolism and increased production of reactive oxygen species, which consequently trigger mechanisms of cellular senescence and apoptotic cell death. Moreover, reduced mitochondrial function can contribute to age-associated disease phenotypes in model organisms and humans. Literature data show beneficial effects of exercise on the impairment of mitochondrial biogenesis and dynamics and on the decrease in the mitophagic capacity associated to aging. Thus, exercise could have effects on the major cell signaling pathways that are involved in the mitochondria quality and quantity control in the elderly. Although it is known that several exercise protocols are able to modify the activity and turnover of mitochondria, further studies are necessary in order to better identify the mechanisms of interaction between mitochondrial functions, aging, and physical activity, as well as to analyze possible factors influencing these processes.

## 1. Introduction

Aging is a natural and inevitable process characterized by a progressive decline of individuals both physically and mentally. With age, there is a gradual accumulation of dysfunctional components that results in the deterioration of different biological functions, which finally increases the risk of death [1]. Autophagy, a catabolic mechanism, involves the degradation of damaged cellular and molecular components through the formation of a double membrane structure known as autophagosome, which fuses with the lysosome to form the autophagolysosome, surrounding the structures that will be degraded [2]. Autophagy can be classified according to its selective organelle. Thus, when the target is the mitochondria, it is called mitophagy; ribophagy for the ribosomes; peroxyphagy for the peroxisomes; or reticulophagy for the endoplasmic reticulum, among others [3]. Moreover, it is important to highlight that, although appropriate levels of autophagy induction are required to extend lifespan, a malfunctioning of autophagy occurs in many old organs and tissues [4].

Aging particularly affects mitochondrial homeostasis [5], and it is clear that mitochondria could contribute to aging due to its key role in the complex balance of cellular processes [6]. Age-related changes also affect mitochondrial membrane potential (ΔΨm) [7], inducing the opening of permeability transition pores in the mitochondrial membrane, which leads to mitochondrial depolarization, that is, a decrease in the ΔΨm to −100 mV [8]. Moreover, the age-dependent decline of mitophagy hinders the elimination of dysfunctional or damaged mitochondria and alters mitochondrial biogenesis, leading to a progressive accumulation of mitochondria. These effects result in the deterioration of cellular function [9]. Therefore, physiological aging has been associated with decreased mitophagic processes but also with impaired mitochondrial functions.

It is important to highlight that many interventions leading to health improvement and extension in lifespan, such as calorie restriction, or treatment with rapamycin, spermidine, metformin, or the antioxidant resveratrol, also induce an activation of the autophagic/mitophagic machinery [4]. In addition, regular physical activity has demonstrated benefits on adults' health and has been also identified as an inducer of autophagy *in vivo* [2, 10]. In the same line, recent evidences have demonstrated that exercise is also able to affect the activity and turnover of mitochondria by increasing biogenesis and mitophagy [11].

In this review, we summarize the role that mitochondria, including its biogenesis, dynamics, and mitophagy, play in the aging process, and how modulation of those functions contributes to the mitochondrial adaptations to physical exercise in the elderly.

## 2. Mitochondria and Aging: Functions and Importance

Mitochondria are the powerhouses of the cell, generating a large part of cellular ATP. Moreover, mitochondria are involved in calcium metabolism, contribute to the formation of intracellular reactive oxygen species (ROS), and play a leading role in the initiation of apoptosis, being therefore the key in maintaining cellular homeostasis and acting as important signaling organelles in different tissues [12]. The size and total number of mitochondria depend on the metabolic needs, the state of differentiation, and the different physiological conditions of the cell [13].

**Figure 1:**
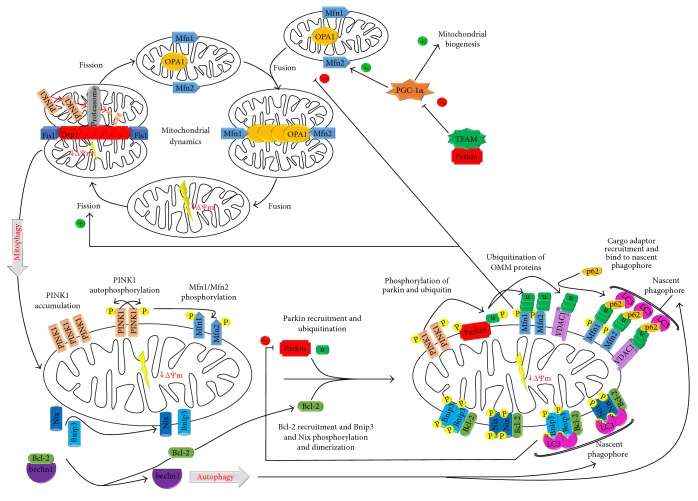
Mitochondrial biogenesis, dynamics, and mitophagy process. Machinery regulating mitochondrial morphology dynamics can regulate mitophagy initiation, so mitophagy is inhibited by fusion process, mediated via Mfn1/2 and OPA1, and promoted by fission process, mediated via Drp1. Fission process generates polarized mitochondria, which is driven to fusion process, and depolarized mitochondria, which is targeted by mitophagy. In polarized mitochondria, PINK1 is imported into IMM and degraded via proteasome. In depolarized mitochondria, PINK1 is accumulated in OMM. There, PINK1 recruits Parkin from the cytosol by Mfn1 and Mfn2 phosphorylation and phosphorylates ubiquitin and ubiquitin-like domain of Parkin. Then, Parkin ubiquitinates OMM proteins, such as VDAC1, which recruit the p62/SQSTM1 autophagy cargo adaptor. This receptor simultaneously binds to LC3 localized in the nascent phagophore. Furthermore, mitochondrial biogenesis can promote the fusion process, blocking mitophagy, through PGC-1*α*, which stimulates Mfn2 expression; and mitophagy can inhibit mitochondrial biogenesis via Parkin, whose association with TFAM inhibits the expression of PGC-1*α*. On the other hand, upon expression, Bnip3 and Nix bind Bcl-2, broking the beclin1/Bcl-2 interaction, so that beclin1 can induce autophagy initiation. Moreover, Bnip3 and Nix are phosphorylated and form homodimers, which integrate in OMM and then bind to LC3. In both cases, the LC3 bond triggers mitochondria to autophagy.

To maintain a healthy status, mitochondria regulate their biogenesis and engage in several dynamic behaviors. The key elements are depicted in Figure 1(). Mitochondrial biogenesis is defined as the coordinated regulation between nuclear gene expression, protein import and transcription of mitochondrial DNA (mtDNA) [14]. This process is regulated by a several transcription factors, such as mitochondrial transcription factor A (TFAM) and the peroxisome proliferator-activated receptor gamma coactivator 1-alpha (PGC-1*α*), as well as at a posttranscriptional level [15]. PGC-1*α* is also involved in other functions, such as control of mitochondrial genome copy number, regulation of mitochondrial dynamics and modulation of oxidative phosphorylation [16]. Moreover, these organelles are highly dynamic and undergo fusion (the joining of two organelles into one) and fission (the division of a single organelle into two) [17]. Fusion process involves three GTPases; mitofusin (Mfn) 1 and Mfn2, which mediate outer mitochondrial membrane (OMM) fusion, and optic atrophy protein (OPA) 1, which regulates inner mitochondrial membrane (IMM) fusion. On the other hand, fission process is mediated by GTPase dynamin-related protein 1 (Drp1) and generates a polarized and a depolarized mitochondrion [18]. Mitochondria also undergo other dynamic behaviors, such as transport (directed movement within a cell) and degradation (targeted destruction via the mitophagic pathway). All these processes are essential for maintaining a healthy mitochondrial population [17].

The maintenance of mitochondrial structural integrity, biogenesis and function is essential to the cells, since mitochondrial dysfunction can induce disturbances in energy metabolism, increase ROS production and, consequently, trigger mechanisms of apoptotic cell death [12]. Moreover, during the last decades, multiple lines of evidence in model organisms and humans have demonstrated that impaired mitochondrial function can contribute to the aging process, as well as age-associated diseases [19, 20]. In fact, it has been shown that decreased mitochondrial performance is a hallmark of aging possibly due to the central role of mitochondria in metabolism and cellular function. Thus, the potential toxicity of mitochondrial ROS (mtROS), originating from mitochondrial respiratory chain, led to the formulation of the oxidative stress theory of aging, which suggested that the accumulation of oxidative damage to macromolecules is an important point in the aging process [21]. Mitochondrial DNA has two characteristics that make it a key target of mtROS: on the one hand, its proximity to the respiratory chain and, on the other, the lack of protective histones. Damaged mitochondrial DNA alters the respiratory chain, increasing the free radical generation and triggering a vicious cycle. These changes result in organic dysfunction and aging phenotype [22]. Recently, however, in contrast to the original theory favoring oxidative damage as a cause for mtDNA mutations and corresponding declines in mitochondrial function, there are strong data arguing that most mammalian mtDNA mutations originate as replication errors made by the mitochondrial DNA polymerase [6, 19].

Additionally, the protein quality control, or proteostasis, plays an important role in age-related decline driving to the accumulation of misfolded and unfolded proteins and to the mitochondrial dysfunction. To solve this problem, and promote cell survival and organism longevity, cells activate a transcriptional response known as mitochondrial unfolded protein response (mtUPR) [23]. In response to the accumulation of unfolded proteins or dysfunctional oxidative phosphorylation system (OXPHOS), cells activate the mtUPR to recover mitochondrial function through the stabilization of mitochondrial protein-folding environment and upregulation of a cytosolic source of ATP production [24, 25]. In addition, to compensate for the OXPHOS activity reduction, mtUPR can induce mitochondrial biogenesis [25]. Finally, if despite mtUPR activation the cell cannot maintain a ΔΨm, the mitochondria are directed to the mitophagy pathway [26].

In aging, skeletal muscle mass decreases from midlife onwards. In addition, mitochondrial functional capacity and mitochondrial density are also reduced [27–29]. However, it is unclear whether these changes are a direct consequence of aging per se, or are due to inactivity. Indeed, a significant improvement in mitochondrial function and an increase in PGC-1*α* expression have been observed in old trained subjects [30, 31]. The expression of deleterious mtDNA mutations within cells seems to be also controlled by the balance between fusion and mitochondrial fission. While fusion may allow the “dilution” of a mutant mtDNA species in a set of wild-type molecules, reducing the functional effect, fission may lead to the removal of aberrant mitochondria via selective mitophagy [6]. Thus, the polarized mitochondrion can be triggered to the fusion process and depolarized mitochondrion is targeted by mitophagy. In this way, mitophagy is promoted and inhibited by fission and fusion events, respectively [18]. Moreover, mitochondrial dynamics also regulates mtUPR and mitochondrial biogenesis, since Mfn2 overexpression causes increased mitochondrial membrane potential, glucose oxidation, and increased expression of nuclear-encoded subunit OXPHOS complexes I, IV, and V, while Mfn2 depletion represses the expression of complexes I, II, III, and V (a subunit). In addition, PGC-1*α* promotes Mfn2 expression [20]. Finally, the balance between biogenesis and degradation plays a central role in the regulation of overall mitochondrial mass. Therefore, the mitophagic machinery seems to be the key in the clearance of the excess of mitochondria or of those old or defective mitochondria [17]. Thus, these mechanisms of cell destruction are activated in order to preserve the health and good working of tissues, since the increase of oxidative stress in tissues could cause DNA damage and mutations that accelerate the aging of the tissue and decrease its longevity [11].

## 3. Mitophagy Mechanisms

Since mitochondria are involved in both adaptive metabolism and survival in response to cellular stress, it is necessary to maintain good mitochondrial functioning through a tight mitochondrial quality control [32]. Recently, mitophagy has gained importance because the damage accumulated in the mitochondria may result in a large number of cell consequences. This process of dysfunctional mitochondria removal occurs by two major pathways, damage-induced mitophagy and developmental-induced mitophagy [13]. Mitophagy not only clears dysfunctional mitochondria but also participates in adaptive response to nutrient deprivation, hypoxia or developmental signals, promoting a reduction in the overall mitochondrial mass [32].

Damage-induced mitophagy has the function of removing damaged/defective mitochondria. It is driven by two main proteins: PTEN-induced putative kinase (PINK), which can sense mitochondrial polarization state, and the E3 ubiquitin protein ligase (Parkin) (Figure 1()). In normal conditions, where mitochondria are polarized, PINK1 is maintained in low basal levels. For that, PINK1 is imported into IMM and processed by mitochondrial peptidases, such as the protease presenilin-associated rhomboid-like protein (PARL) [16]. However, in damaged/defective mitochondria, which are depolarized, this process is inactivated since ΔΨm is insufficient to transfer PINK1 to IMM, accumulating at the OMM [33, 34]. There, some mitochondrial proteins are phosphorylated by PINK1, which results in the recruitment of autophagy cargo adaptors that bind to the autophagosome through light chain 3 protein (LC3) and, subsequently, to the lysosome. Thereby, mitochondria are degraded by autophagolysosomes [32]. To amplify this signal, PINK1 recruits Parkin from the cytosol by Mnf1 and Mnf2 phosphorylation. In addition, PINK1 phosphorylates ubiquitin and ubiquitin-like (Ubl) domain of Parkin, which activates Parkin E3 ligase activity and facilitates its recruitment to OMM [18].

Once on the OMM, Parkin ubiquitinates OMM proteins, in addition to ubiquitinating itself [35]. First, Parkin can trigger the ubiquitination of Mfn1 and Mfn2, preventing the fusion of the mitochondria and working as a mechanism of isolation when these organelles are damaged, for subsequent sequestration and degradation by selective autophagy [36]. Then, Parkin ubiquitinates OMM proteins, such as voltage-dependent anion channel (VDAC) 1, generating polyubiquitin chains via Lys27, so that recruits LC3-interacting region- (LIR-) containing autophagy receptors, such as sequestosome (p62/SQSTM), which simultaneously binds to LC3, pointing to the mitochondria that should be eliminated [13, 18]. Additionally, Parkin regulates mitochondrial biogenesis through its association with TFAM, which inhibits the expression of PGC-1*α* as well as its target gen, the nuclear respiratory factor (NRF) 1 [15].

Developmental process-induced mitophagy has the function of programmatic elimination of excessive mitochondrial population [13]. This type of mitophagy is driven by two main proapoptotic proteins, a member of the B-cell lymphoma 2 family (Bcl-2), adenovirus E1B 19 kDa-interacting protein (Bnip) 3 and Nix (Nip3-like protein X or Bnip3L), which induce mitophagy by means of several potential mechanisms [37]. However, mitophagy mediated by Bnip3/Nix is different from the PINK1/Parkin pathway, since PINK1 and Parkin proteins cannot bind directly to autophagosome receptors, while Bnip3 and Nix directly bond the autophagy machinery components (Figure 1()) [38].

Bnip3 can induce autophagy through several mechanisms, including mitochondrial depolarization, mitochondrial permeability transition pore (MPTP) aperture, or interference in fission-fusion machinery [39]. Phosphorylation regulates mitophagy activity of Bnip3. This phosphorylation promotes binding to LC3II and Golgi-associated ATPase enhancer of 16 kDa (GATE-16), and phosphorylation of serine residue 24 increases the affinity for them. On the other hand, phosphorylation at residues at its C-terminus, adjacent to the transmembrane domain, decreases the mitochondrial damage generated by Bnip3 and inhibits cell death but does not block autophagy/mitophagy. Additionally, Bnip3 increases the location of Drp1 to mitochondria [40].

Nix is already required for programmed mitophagy during maturation of reticulocytes, which includes the removal of membrane organelles, such as mitochondria, through a process related to autophagy, with the main difference that the content of the autophagy vacuole is not recycled but eliminated by exocytosis [39]. It is possible that Nix induces mitophagy by its interaction with LC3. LC3 interacts with gamma-aminobutyric acid receptor-associated protein (GABARAP), to form the LC3/GABARAP complex, and mediates mobilization of autophagosome to mitochondria to be eliminated [18, 36]. In addition, Nix can also interact with protein encoded by the BECN1 gene (beclin1)/Bcl-2 complex to release beclin1, thus freeing beclin1 to induce autophagy [13, 37].

Other phospho-regulated OMM-localized mitophagy receptors are FUN14 domain containing (FUNDC) 1 and Bcl-2-like protein 13 (Bcl2-L-13)/Bcl-Rambo, which promote mitophagy through directly binding to components of the autophagy machinery [38]. Furthermore, OMM-localized lipids, such as ceramide, a sphingolipid, or cardiolipin, a dimeric phospholipid, can also recruit mitophagic machinery binding directly to LC3 [18, 41].

## 4. Effects of Aging on Mitochondrial Function and Mitophagy

A proper working maintenance of both mitochondrial quantity and quality is strictly related to the conservation of an adequate concentration of several proteins, such as PGC-1*α*, TFAM, OPA1, Drp1, Mfn1, Mfn2, mitochondrial fission (Fis) 1 protein, PINK1, Parkin, VDAC1, Bnip3, and Nix, which are involved in mitochondrial biogenesis, dynamics and mitophagy process. However, organic alterations, characteristic of aging, can modify the concentration of these proteins, destabilizing their functions (Table 1).

Some studies that relate autophagy and/or mitochondrial function to the aging process show results that may be different [10, 42–44]. Specifically, regarding proteins involved in biogenesis, most studies found a decrease in mRNA and protein expression of PGC-1*α* [45–53]. These data indicate that, during aging, the reduced mitochondrial biogenesis may be due to the lack of response of PGC-1*α* to different stimuli. In fact, aged rats behave as PGC-1*α* knockout mice [54]. On the contrary, TFAM, another transcription factor that regulates mitochondrial synthesis, seems to be increased in the muscle and brain from old rats and decreased in the liver or in the muscle from very old rats [48, 51, 55–57]. In humans, TFAM decreases or does not change significantly in the skeletal muscle [49, 58]. Those results seem to indicate that several tissue-specific mitochondrial changes associated with aging might be due to a fine modulation of TFAM binding to mtDNA.

OPA1 is located on the IMM and regulates mitochondrial fusion and ridge structure. In addition, OPA1 can control mitophagy by promoting the stabilization of mitochondrial ridges, which, in turn, act against mitochondrial dysfunction and excessive production of ROS. Thus, a higher expression of this protein indicates an increase in mitochondrial fusion and a decrease in mitophagy [33]. Most research seems to indicate an increase of this protein in the muscle and brain of aged rodents [44, 59–61]. OPA1 acts as a mitochondrial fusion factor, perhaps by interacting with mitofusins or other outer membrane fusion factors [62, 63]. In this line, Mfn1 and Mfn2 also increased in mouse and rat muscles during senescence [43, 44, 48, 59–61]. However, other studies point to an absence of changes or even decreases in these proteins responsible for mitochondrial fusion [30, 45, 46, 58, 60, 64–66]. On the other hand, and as previously mentioned, mitophagy is promoted by fission [18]. Most authors have shown a decrease in Drp1 levels in the heart, brain, and muscle from old rodents [59–61, 67]. Moreover, the ratio between Mfn2 and Drp1, an index of the balance between fusion and fission processes, was significantly increased in atrophied skeletal muscle of aged mice [60]. These results reinforce the possibility that OPA1 might act as an inhibitor of mitochondrial fission [64]. However, results about the role of Fis1 protein in the aging process are not conclusive [30, 44, 45, 48, 58, 59, 65–67].

PINK1 expression seems to be diminished in hepatocytes and liver tissue from mouse over 18 months of life [65], but in samples from human and rat muscles, no significant changes associated with age have been found [59, 68]. Other authors have reported an increase in muscle Parkin levels of old rats, which might be a further consequence of lipofuscin accumulation within lysosomes, resulting in a buildup of cellular debris [44]. Moreover, the dysregulation of mitochondrial dynamics with aging leads to the accumulation of unhealthy mitochondria, which could result in the recruitment of Parkin in order to control mitochondrial quantity and quality [69].

VDAC1 determines the permeability and conductance of the outer membrane and plays an important role as a modulator of permeability of membrane pores [33]. It has been demonstrated that the level of VDAC1 is significantly decreased in mitochondria isolated from the brain of old rats [70]. The lower protein content might be related to the changes in the mitochondrial susceptibility to Ca^2+^ overloading and for the permeability of membrane pore facilitation found with aging [70]. The same result was observed in triceps muscle from old rats [59]. However, other studies note that, apparently, aging does not decrease the expression of this protein in human muscles when young and old are compared [68, 71].

Regarding the effect of aging on the expression of Bnip3, literature does not seem to be very conclusive. Some studies have been found that this protein may increase in the muscles of sedentary elderly [64, 72]. A possible explanation may be that Bnip3 induction compensates for the loss of mitochondrial autophagy and minimizes mitochondrial damage [64]. Conversely, Bnip3 was decreased in the heart of old rats [67] whereas in the muscle of aged humans it remained unchanged [66, 68]. The effects of aging on Nix, and subsequently on autophagy and mitochondrial clearance, in the skeletal muscles are scarcely known. It has been reported that the expression of Nix increases in the skeletal muscles of old mice [73]. This observation, while consistent with research that shows an increase in autophagy markers in aged muscles [44], contrasts with others authors that have reported aging-associated decline in mitophagy [9, 74]. Perhaps, the explanation for this discrepancy may be the extreme ages (3–5 months old versus 22–24 months old) of mice used in the study by Ko et al. [73]. Taking into account these data, it is possible to infer that the increase of both Bnip3 and Nix, associated with age, may indicate a failure in the mechanism of mitophagy. Possibly, Bnip3 and Nix accumulation could hinder these proteins to perform their function in the mitophagic process.

The autophagy markers beclin1 and LC3 (LC3II or LC3II/I) also seem to be altered in the aging process. Thus, beclin1 levels have been reported to decrease in human and mouse muscles [43, 66] and in peripheral blood mononuclear cells (PBMCs) from old subjects [10]. However, other studies in human and rat muscles did not show any change [44, 68, 75]. These differences could be due to the tissue type, as observed in gastrocnemius and triceps rat muscles [59]. Several studies have shown that LC3II or LC3II/LC3I increased in mouse aged muscle [64], while other studies have described no changes in human and rat aged muscle [44, 66]. On the other hand, previous results from our research group showed that the LC3II/I ratio was markedly lower in mononuclear cells from old subjects when compared to a young group [10]. A similar phenomenon has been also observed by others when young and older adult skeletal muscles were studied [75].

Overall, it has been reported that aging is associated with a decline in the mitochondrial function, in the accumulation of abnormal mitochondria and in the mitophagic capacity of the organism. However, studies analyzed do not exhibit a consensus in relation to most proteins involved. It is up to future research to determine more precisely the mechanisms of interaction between aging and mitochondrial functions, as well as to analyze possible factors that could influence this process, such as sex, nutritional pattern or habitual level of physical activity.

## 5. Effects of Exercise on Mitochondrial Biogenesis, Dynamics, and Mitophagy in Aging

Physical exercise has been proposed as a nondrug treatment against different diseases for people of all ages [76]. In addition, it is suggested that regular exercise could promote an increase in mitophagy capacity [14] and produce effects on the mitochondrial life cycle (Table 2).

Theoretically, physical exercise could also have effects on the major signaling pathways that are involved in the quality and quantity control of mitochondria during the aging process, such as mitophagy [77]. Mitochondria produce ROS that can act as signaling molecules, inducing a survival response or causing damage to cellular components. However, contraction of the skeletal muscle during physical exercise can activate a mitochondrial response that improves the quality of mitochondria in different ways: (1) increasing biogenesis; (2) enhancing the expression and action of the proteins involved in the mitochondrial dynamics, as OPA1; (3) raising mitochondrial turnover by the action of mitophagy proteins, such as PINK1, Parkin, Nix and Bnip3; and (4) increasing the quality control of mitochondria through the degradation of damaged or dysfunctional mitochondria [78].

In this regard, exercise would produce an immediate increase of mitochondrial activation involving a significant increase in the expression of the transcription factor PGC-1*α* and of nuclear genes encoding mitochondrial protein expression. All these modifications would result in a higher mitochondrial content, with better performance, such as raised oxygen consumption and ATP synthesis, reduced ROS production, and increased mitophagy capacity [31]. In fact, independent of the volume and intensity, regular endurance physical exercise induces the gene expression of PGC-1*α* in the skeletal muscles from old human and rodents [30, 31, 47, 48, 79]. Even 7 days of electrostimulation [80] or a single bout of high intensity exercise, both endurance and resistance [68, 81, 82], are able to increase this transcription factor, at mRNA level, in aged rat and human muscles [80]. Only in one study, based on a single bout of 45 min of moderate endurance exercise plus vigorous exercise until exhaustion, no changes in muscle PGC-1*α* levels were observed [58]. In the same line, TFAM mRNA increased in the muscle after several weeks of aerobic exercise [31, 47] but no changes were detected in short trainings (3–6 weeks) [48, 83] or a single bout of exercise [58] carried out by old humans or rats.

OPA1 is another protein that may be unchanged in response to voluntary exercise [46, 84]. However, it has been also demonstrated that 12 weeks of aerobic exercise increased OPA1 mRNA content in the skeletal muscle of old subjects [85]. The mechanism by which exercise could promote the increase of this protein is not established, but it seems to be related to the maintenance of higher levels of mitochondrial fusion and fission processes during exercise [86]. Although results are not conclusive, the increase showed in muscle Mfn1 mRNA during aging could be reversed by a single bout of running in humans [58] or 6 weeks of treadmill running training in rats [48]. However, other studies in human muscle demonstrated that Mfn1 and Mfn2 were unaltered in response to exercise [45, 46, 83, 84, 87]. On the other hand, although Mfn1 and Mfn2 were increased after 12 weeks of cycling [30], a similar aerobic protocol, with the same duration and higher intensity, did not show an increase in the Mfn1 and Mfn2 mRNAs [85]. The role of Drp1 and Fis1 is also contradictory in response to different types of exercise in the elderly [30, 45, 46, 48, 58, 83, 85, 87]. This fact was also described in the previous section when only the effect of the age was evaluated.

In relation to the proteins involved in mitophagy, it is possible to consider that a decrease in the bioavailability of PINK1 and Parkin1 may indicate a higher degree of mitochondrial dysfunction during aging [87]. Thus, the increase of these proteins, caused by exercise, may be an attempt to promote the control of mitochondrial quality through the action of the mitophagy machinery [69]. However, there is no general consensus on the effects of exercise during aging. Studies that evaluated PINK1 found that this protein may not suffer alterations in human muscles [68, 85, 87], suggesting that this pathway is not stimulated by exercise. On the other hand, muscle Parkin protein could remain unchanged [68, 85] or increase at the level of mRNA [87] in response to exercise practice in the elderly. It is important to highlight that, in those studies where no significant changes were observed in PINK1 or Parkin, there was a trend towards an increase in the expression of both proteins [68, 85]. However, results from those studies must be taken with caution due to several reasons. For example, in the study by Ogborn et al. [68], it is remarkable that both, young and old, were grouped to demonstrate the effect of a single bout of resistance exercise over time, while Fealy et al. [85] recruited only obese old subjects (body mass index: 34.6 kg/m^2^), who carried out an aerobic training during 12 weeks.

VDAC1, another Parkin-ubiquitinated protein, appears to exhibit no alterations in response to exercise. Thus, 20 sessions of aerobic cyclergometer training did not change muscle VDAC1 in old participants [71]. A single bout of resistance exercise also did not modify the protein content of VDAC1 [68]. So, further studies are needed to analyze the behavior of this protein in relation to the practice of physical exercise, to determine more precisely whether physical exercise can induce alterations in its expression.

Nix mRNA increases in the muscle sample taken from the vastus lateralis after ultraendurance exercise in human adults [88]. This protein exerts a direct regulation of autophagy, as an increase in its expression may indicate a higher formation of autophagosomes and greater mitophagy flux. In this way, it seems that exercise may stimulate the increase of Nix mRNA and, consequently, of mitophagy, although the mechanism by which exercise produces this increase is not clarified [88]. On the other hand, no changes are found in Nix mRNA in response to exercise in the muscle from aged subjects after a single bout of resistance training [68].

Bnip3 is another protein with no clear behavior in response to exercise. In fact, in two studies carried out in the skeletal muscle of active elderly without exercise intervention, opposite results were obtained (increase and decrease, resp.) [72, 87]. Moreover, no changes were found in Bnip3 mRNA after 24 weeks of combined exercise [89], or after a single bout of resistance training [68]. These contradictory responses could be due to methodological differences, because different exercise protocols were used.

Most of the researches carried out in the samples from human muscles seem to point that the level of physical activity does not change beclin1 content in old subjects [72]. In the same line, beclin1 remains unchanged in response to a single bout of resistance training [68] or to 8 weeks of strength training [75]. Nevertheless, an increase of mRNA beclin 1 level in the muscle from elderly individuals who practiced physical activity has been demonstrated [87]. The same results were obtained by our research group when analyzing the protein content in peripheral blood mononuclear cells, after 8 weeks of aerobic or resistance training [2, 10]. On the other hand, LC3II or LC3II/I levels were increased in human muscle and mononuclear cells after a single bout of resistance training [68] or 8 weeks of aerobic or high intensity resistance training [10, 75]. Likewise, a training program of 8 weeks of resistance training induced a nonsignificant increase in the LC3II/LC3I ratio in PBMCs from elderly participants [2]. Finally, brain levels of the autophagy marker LC3II were not significantly altered in the mouse after completing 3 weeks of treadmill running [83].

Joint analysis of all studies does not allow establishing a clear trend on the effect of physical activity in relation to the type of exercise, volume, or intensity. Overall, aerobic or combined chronic long-term training (more than 12 weeks) seems to be the type of exercise most effective to counteract the age-related damage at the mitochondrial level [30, 31, 47, 85, 89]. Therefore, it seems that exercise stimulates biogenesis, dynamics, and mitophagic capacity, although there is not a consensus about the behavior of different proteins in response to a physical stimulus. Future studies should focus on investigating the effects that different protocols of physical exercise can cause on the expression of mitochondrial proteins, as well as whether exercise practice could prevent mitochondrial damage and the effects on the dysfunctional mitochondria.

## 6. Conclusions

The maintenance of mitochondrial function and mitophagy is essential to the cells, since mitochondria are involved in both adaptive metabolism and survival in response to cellular stress. Although the studies analyzed do not exhibit a general consensus, it seems that aging impairs mitochondrial biogenesis and dynamics and decreases the mitophagic capacity of the organism. Several interventions, such as any type of physical exercise, are able to affect the activity and turnover of mitochondria by increasing biogenesis (specifically PGC-1*α* and TFAM). In addition to, the changes detected in the biogenesis, aerobic, or combined long-term training also seem to produce increases in several markers of mitochondrial dynamics and mitophagy. However, we consider that it is very important to assess all these markers in different exercise protocols in order to establish a direct relationship between the detected changes and type, intensity, and volume of exercise. So, further research is necessary to determine the mechanisms of interaction between mitochondrial functions, aging, and physical activity, as well as to analyze possible factors that are supposed to influence these processes.

## Figures and Tables

**Table 1:** Effect of aging on mitochondrial proteins.

Reference	Tissue	Subject	Comparative	Results
[58]	Muscle	Humans	Sedentary and active youths and elderly	NS (mRNA): PGC-1*α*; TFAM; Fis1; Mfn1
[45]	Carotid arteries	Mouse	Sedentary and active youths and elderly	↓: PGC-1*α*; Mfn2
				↑: Fis1
[46]	Muscle	Humans	Youths/elderly	↓: PGC-1*α*; OPA1
				NS: Mfn2; Drp1; Fis1
[47]	Muscle	Rats	Youths/elderly	↓ (mRNA and protein): PGC-1*α*; TFAM
[73]	Muscle	Mouse	Youths/elderly	↑: Nix; LC3
[48]	Muscle	Rats	Youths/elderly	↓: PGC-1*α*
				↑: TFAM; Fis1; Mfn1
[30]	Muscle	Humans	Youths/elderly	NS: PGC-1*α*; Mfn1; Mfn2; Fis1
[49]	Muscle	Humans	Youths/elderly	NS: PGC-1*α*; TFAM
[68]	Muscle	Humans	Youths/elderly	NS (mRNA): PGC-1*α*; TFAM; beclin1; Bnip3
				NS: Parkin; PINK1; VDAC1
[50]	Muscle	Humans	Youths and active and sedentary elderly	↓: PGC-1*α*
[72]	Muscle	Humans	Youths and active and sedentary elderly	↑: PGC-1*α*; beclin1 (elderly versus youths); Bnip3 (sedentary elderly versus youths)
				NS: Bnip3 (active elderly versus youths)
[71]	Muscle	Humans	Youths/elderly	NS: VDAC1
[43]	Muscle	Mouse	Youths/elderly	↓: beclin1; Fis1
				↑: Mfn1; Mfn2
				NS: PGC-1*α*; TFAM; OPA1; Drp1; LC3II
[67]	Heart	Rats	Youths, old adult, and senescent	↓: Bnip3; Drp1; Fis1
				↑: OPA1 (old adults versus youths); Mfn2; LC3I
				NS: beclin1; LC3II
[59]	Gastrocnemius (G) and triceps (T) muscle	Rats	Youths/elderly	↓ (G): Drp1; Fis1; beclin1; LC3II; (T): PINK1; VDAC1
				↑ (G): OPA1; Mfn1; VDAC1; (T): OPA1; Mfn1; Drp1; Fis1
				NS: (G): PINK1; (T) beclin1; LC3II
[51]	Muscle	Rats	Youths/elderly	↓: PGC-1*α*
				↑: TFAM
[55]	Cerebellum, heart, kidney, and liver	Rats	Youths/elderly	↑: TFAM
				NS (heart): TFAM
[66]	Muscle	Humans	Youths, middle-aged, and elderly	NS: OPA1; Mfn2; Fis1; Drp1; Bnip3; beclin1; LC3II/I
[60]	Muscle	Mouse	Youths/elderly	↑: Mfn2/Drp1
				NS: OPA; Drp1; Mfn1; Mfn2
[44]	Muscle	Rats	Youths/elderly	NS: Drp1; beclin1; LC3II
				↑: Parkin; Fis1; OPA1; Mfn2
[56]	Brain	Rats	Youths/elderly	↑: TFAM
[52]	Liver	Rats	Middle-aged, old (18 m), and very old (28 m)	↓: TFAM; PGC-1*α*
				NS: VDAC
[57]	Brain, muscle, and liver	Rats	Middle-aged, old (18 m), and very old (28 m)	↑ (brain): TFAM
				↓ (muscle and liver): TFAM
[64]	Muscle	Mouse	Youths/elderly	↑: Bnip3; LC3II; LC3II/I
				↓: Mfn2
				NS: LC3I
[61]	Brain	Mouse	Youth, middle-aged, and elderly	↓: TFAM; Drp1; Mfn2
				↑: OPA1; Mfn1
[53]	Heart	Humans	Youths/elderly	↓: PGC-1*α*
[75]	Muscle	Humans	Youths/elderly	↓: LC3II/I
				NS: beclin1
[10]	PBMCs	Humans	Youths/elderly	↓: LC3II/I; beclin1

**Table 2:** Effect of exercise on mitochondrial proteins in aging.

Reference	Tissue	Subject	Comparative	Training	Results
[58]	Muscle	Humans	Sedentary and active youths and elderly	Single bout of treadmill running of 45 min at 70–75% VO_2max_ plus exercise until exhaustion at 90% VO_2max_	↓ (mRNA): Fis1; Mfn1
					NS: PGC-1*α*; TFAM
[31]	Muscle	Humans	Sedentary elderly and active elderly Sedentary elderly + training	16 weeks of aerobic exercise (bike, walk, run, or row), 3 times/week at 75–80% HR	↑ (mRNA): PGC-1*α*, TFAM (sedentary elderly + training)
					NS (mRNA): PGC-1*α*, TFAM (sedentary elderly versus active elderly)
[81]	Muscle	Humans	Trained elderly and untrained elderly Untrained elderly + acute exercise	Single bout of high intensity interval exercise on a bicycle ergometer for 20 min at 80% of peak power output	↑ (mRNA): PGC-1*α* (untrained + acute exercise)
					↑: PGC-1*α* (trained versus untrained elderly)
					NS: PGC-1*α* (untrained + acute exercise)
[87]	Muscle	Humans	Sedentary elderly Active elderly	Without exercise intervention	↑ (mRNA): Bnip3; Drp1; Parkin; beclin1
					NS (mRNA): Mfn2; PINK1; LC3
					NS: beclin1
[45]	Carotid arteries	Mouse	Sedentary and active youths and elderly	10 weeks of voluntary aerobic exercise in a wheel running	NS: PGC-1*α*; Fis1; Mfn2
[83]	Brain	Mouse	Elderly	3 weeks of treadmill running for 60 min at 15–19 m/min and 10° incline	NS (cortex): PGC-1*α*, TFAM, Mfn2; LC3II
					NS (striatum): PGC-1*α*, TFAM, Drp1, Mfn2; LC3II
					↑ (cortex): Drp1
[82]	Muscle	Humans	Elderly	Single bout of bicycle exercise at 75% VO_2max_ until exhaustion	↑ (mRNA): PGC-1*α*
					NS (mRNA): TFAM
[46]	Muscle	Humans	Youths/elderly	Without exercise intervention	NS: PGC-1*α*; TFAM; Mfn2; OPA1; Drp1; Fis1
[47]	Muscle	Rats	Youths/elderly	12 weeks of treadmill running 5 times/week for 45 min at 17.5 m/min and 10° incline	↑: PGC-1*α*; TFAM
					NS (mRNA): PGC-1*α*; TFAM
[48]	Muscle	Rats	Youths/elderly	6 weeks of treadmill running for 60 min at 10–22 m/min and 5–10% incline	↑: PGC-1*α*
					↓: Mfn1
					NS: TFAM; Fis1
[30]	Muscle	Humans	Youths/elderly	12 weeks of cycling 3-4 times/week at 60–80% HR reserve for 20–45 min	↑: PGC-1*α*; OPA1; Mfn1; Mfn2; Fis1
[49]	Muscle	Humans	Youths/elderly	Without exercise intervention	↑: PGC-1*α*; TFAM
[80]	Muscle	Rats	Youths/elderly	7 days of electroestimulation 3 h/day at 10 Hz for 0.1 ms duration	↑: PGC-1*α*; TFAM
[68]	Muscle	Humans	Youths/elderly	Single bout of resistance training (4 sets of rept. at 75% 1RM with 2 min rest between sets)	↑ (mRNA): PGC-1*α* (3 h); TFAM (24 h); LC3II (3 h)
					↑: LC3II (48 h)
					NS (mRNA): Nix; Bnip3
					NS: Parkin; PINK1; VDAC1; beclin1
[79]	Muscle	Mouse	Youths/elderly	6 weeks of voluntary aerobic exercise in wheels running	↑: PGC-1*α*
[84]	Muscle	Mouse	Elderly	Single bout of voluntary aerobic exercise in wheels running for 3 h	NS: OPA1; Mfn2
[72]	Muscle	Humans	Youths and active and sedentary elderly	Without exercise intervention	↓: Bnip3
					NS: PGC-1*α*; beclin1
[85]	Muscle	Humans	Elderly	12 weeks of aerobic exercise for 60 min (20 min cycle ergometer and 40 min treadmill walking) at 80–85% HR_max_ 5 days/week	↑ (mRNA): OPA1; Drp1
					↓: phosphorylated Drp1
					NS (mRNA): Mfn1; Mfn2; Fis1; PINK1; Parkin
[71]	Muscle	Humans	Youths/elderly	14 days of immobilization and 20 sessions of aerobic cycle ergometer training with 48–58 min at 85% HR_max_	↑ (immobilization + training versus immobilization): VDAC1
					NS (immobilization + training versus control): VDAC1
[89]	Muscle	Humans	Elderly	24 weeks of combined exercise (walking + strength training + flexibility) 2-3 times/week at moderate intensity	NS (mRNA): Bnip3
					↑ (mRNA): PGC-1*α*; TFAM; LC3II
					↑: TFAM
[75]	Muscle	Humans	Youths/elderly	5 days of bed rest and 8 weeks of high intensity resistance exercise	↑: LC3II/I
					NS: beclin1
[10]	PBMCs	Humans	Elderly	8 weeks of aerobic training	↑: LC3II/I¸beclin1
[2]	PBMCs	Humans	Elderly	8 weeks of resistance training	↑: beclin1
					NS: LC3II/I

## Abbreviations

Bcl-2: B-cell lymphoma 2 family
Bnip3: Adenovirus E1B 19 kDa-interacting protein 3
Bnip3L: Bnip3-like protein
Drp1: Dynamin-related protein 1
FUNDC1: FUN14 domain containing 1
GABARAP: Gamma-aminobutyric acid receptor-associated protein
GATE-16: Golgi-associated ATPase enhancer of 16 kDa
IMM: Inner mitochondrial membrane
LC3: Light chain 3
LIR: LC3-interacting region
Mfn: Mitofusin
MPTP: Mitochondrial permeability transition pore
mtDNA: Mitochondrial DNA
mtROS: Mitochondrial reactive oxygen species
mtUPR: Mitochondrial unfolded protein response
Nix: Nip3-like protein X
NRF1: Nuclear respiratory factor 1
OMM: Outer mitochondrial membrane
OPA1: Optic atrophy protein 1
OXPHOS: Oxidative phosphorylation system
p62/SQSTM1: Sequestosome 1
Parkin: E3 ubiquitin protein ligase
PARL: Presenilin-associated rhomboid-like protein
PBMCs: Peripheral blood mononuclear cells
PGC-1*α:*: Receptor gamma coactivator 1-alpha
PINK1: PTEN-induced putative kinase 1
ROS: Reactive oxygen species
TFAM: Mitochondrial transcription factor A
Ubl: Ubiquitin-like
VDAC1: Voltage-dependent anion channel 1
ΔΨm: Mitochondrial membrane potential.
